# A Novel Point-of-Care Smartphone Based System for Monitoring the Cardiac and Respiratory Systems

**DOI:** 10.1038/srep44946

**Published:** 2017-03-22

**Authors:** Kwanghyun Sohn, Faisal M. Merchant, Omid Sayadi, Dheeraj Puppala, Rajiv Doddamani, Ashish Sahani, Jagmeet P. Singh, E. Kevin Heist, Eric M. Isselbacher, Antonis A. Armoundas

**Affiliations:** 1 Cardiovascular Research Center, Massachusetts General Hospital, Boston, MA, USA; 2 Cardiology Division, Emory University School of Medicine, Atlanta, GA, USA; 3 Cardiology Division, Cardiac Arrhythmia Service, Massachusetts General Hospital, Boston, MA, USA; 4 Healthcare Transformation Lab, Massachusetts General Hospital, Boston, MA, USA; 5 Institute for Medical Engineering and Science, Massachusetts Institute of Technology Cambridge, MA, USA

## Abstract

Cardio-respiratory monitoring is one of the most demanding areas in the rapidly growing, mobile-device, based health care delivery. We developed a 12-lead smartphone-based electrocardiogram (ECG) acquisition and monitoring system (called “cvrPhone”), and an application to assess underlying ischemia, and estimate the respiration rate (RR) and tidal volume (TV) from analysis of electrocardiographic (ECG) signals only. During *in-vivo* swine studies (n = 6), 12-lead ECG signals were recorded at baseline and following coronary artery occlusion. Ischemic indices calculated from each lead showed statistically significant (p < 0.05) increase within 2 min of occlusion compared to baseline. Following myocardial infarction, spontaneous ventricular tachycardia episodes (n = 3) were preceded by significant (p < 0.05) increase of the ischemic index ~1–4 min prior to the onset of the tachy-arrhythmias. In order to assess the respiratory status during apnea, the mechanical ventilator was paused for up to 2 min during normal breathing. We observed that the RR and TV estimation algorithms detected apnea within 7.9 ± 1.1 sec and 5.5 ± 2.2 sec, respectively, while the estimated RR and TV values were 0 breaths/min and less than 100 ml, respectively. In conclusion, the cvrPhone can be used to detect myocardial ischemia and periods of respiratory apnea using a readily available mobile platform.

Given the increasing prevalence of chronic disease in the United States, together with the pressure to curtail health care costs, more efficient and cost effective methods of evaluating and monitoring patients will be essential. At the same time, there is an increased availability of new technologies and an ever-improving health information technology infrastructure. To this end, mobile-health technologies are expected to function not only as monitoring devices, but as essential components in the healthcare delivery^1^, especially among patients with chronic conditions^2^,^3^,^4^,^5^. Indeed, given that almost two-thirds of US adults now have a smartphone^6^, wireless devices have the potential to usher in a new era in medicine and a transition from population-level health care to personalized medicine.

Among the chronic diseases that affect the US population, cardiovascular conditions are among the most prevalent and costly to manage. For example, in the US alone, HF affects nearly 6 million people, that results in an estimated 1 million admissions per year, and costs more than $30 billion per year^7^. Recently, home telemonitoring interventions for patients with HF have led to a reduction in the relative risk of all-cause mortality and HF-related hospitalizations, compared to usual care^5^.

A pattern of Cheyne-Stokes respiration (CSR) has been identified in up to 40% of patients with chronic HF and its presence has been associated with cardiac dysrhythmias^8^,^9^. Additionally, among HF patients CSR is a marker of worse prognosis and increased mortality, whereas a reduction in CSR is marker of a positive response to HF medical therapy^10^. Assessments of respiratory rate (RR) and tidal volume (TV) are important to determining the frequency of CSR in HF^10^, so real-time access to such data could be of great value in clinical practice.

Coronary heart disease is another common and chronic cardiovascular condition that affects more than 15 million Americans^7^. It has been recognized that when managing patients with coronary heart disease, relying on symptoms of angina leads to a significant underestimation of the true frequency of myocardial ischemia, as ischemia is often silent. Therefore ambulatory monitoring of electrocardiographic ST-segment changes has the potential to detect ischemia earlier and, in turn, prompt appropriate interventions sooner, than would be otherwise be possible^11^. Moreover, silent ambulatory ST-segment depression is associated with an increased incidence of coronary events in asymptomatic men, so routine assessment of such ST-segement depression could be used to better stratify populations are risk^12^.

Such clinical observations highlight the potential value of portable home-based devices to monitor respiratory and cardiovascular parameters in ambulatory populations with heart failure and coronary heart disease^13^,^14^,^15^,^16^,^17^,^18^,^19^,^20^. We therefore developed a wireless cardiorespiratory monitoring system based upon just a smartphone and an electrocardiographic (ECG) device that was built using three commercially available components: an ECG module, a microcontroller board, and a Bluetooth module. The smartphone, named “cvrPhone,” estimates the ischemic state of a subject in real-time based upon 12-lead ECG signals that are transmitted from the ECG device. It also estimates the RR and TV based on the respiration-induced periodic fluctuations of ECG signals. In this study, we examine the hypothesis that we can assess coronary ischemia and apneic events using only body surface ECG signals that are recorded and analyzed by the smartphone.

## Methods

### Hardware Architecture

**Figure 1:**
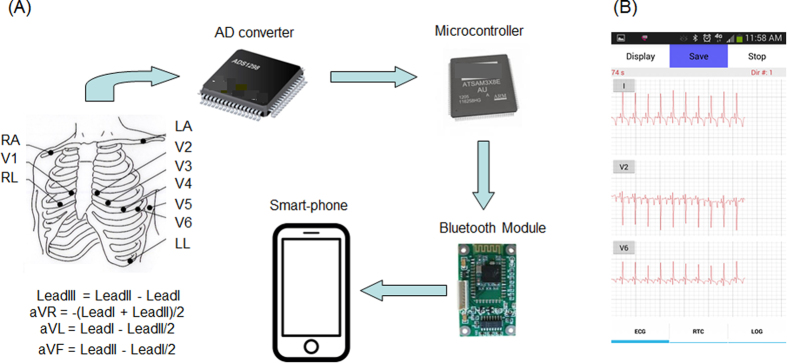
The smartphone-based ECG acquisition system, or “cvrPhone”. The Bluetooth-enabled ECG acquisition system is composed of three commercially available parts: An analog-to-digital (AD) converter, a microcontroller board and, and a Bluetooth module. (**A**) Flow-diagram of the 12-lead ECG signals from the torso to smartphone. Ten electrodes are placed on the torso for the recording of 8 ECG leads (Leads I and II and six precordial leads). The AD converter amplifies and digitizes the 8 ECG leads. Then the signals are transmitted to the smartphone through the HC-05 Bluetooth module, and the remaining leads (Leads III, aVR, aVL and aVF) are calculated. (**B**) Real-time display of selected three ECG signals on the smartphone screen.

The ECG device is composed of an analog-to-digital (A/D) converter (ADS1298, Texas Instruments, Dallas, TX), a microcontroller board (Arduino Due AT91SAM3 × 8E, Atmel, San Jose, CA), and a Bluetooth module (HC-05, Guangzhou HC Information Technology Co., Ltd., Guangzhou, China). The AD converter amplifies and digitizes the analog ECG signal from electrodes (Fig. 1A), and the microcontroller transmits the digitized ECG signal to the smartphone (Fig. 1B). Uninterrupted Bluetooth communication could be achieved up to 10 meters away from the smartphone.

### Microcontroller Software

The microcontroller receives digitized ECG signals from the AD converter and transmits them to the smartphone through Bluetooth at the user’s request. We used the open-source integrated development environment (IDE) Arduino 1.5.4 for the microcontroller programming. There are two main steps in the function of the embedded software: *first*, initialize the AD converter and the Bluetooth module; and *second*, transmit the ECG signals upon user’s request (see Supplement Figures S1,S2).

The settings of sampling rate, gain and reference voltage of the AD converter are 500 samples per second (SPS), 12 and 24 V respectively. The Wilson Central Terminal (WCT, (RA + LA + LL)/3) is used as the reference voltage for the precordial leads. The signal from the AD converter has 24 bit resolution, but it is reduced to 16 bit by dropping the upper and lower 4 bits to reduce the transmission load via Bluetooth. The ECG signal covers ± 12.5 mV with resolution of ~0.38 μV. The baud rate of the Bluetooth module is 115200.

After the initialization, the microcontroller repeats sending ECG signals according to the user’s “actions.” There are three different actions: “save,” “display.” and “stop”: (i) At “save” action, the smartphone displays ECG data on the screen, saves the ECG data, and calculates the ischemic index, RR and TV in real-time. The microcontroller transmits the ECG signals at 500 SPS to the smartphone for the “save” action. (ii) At “display” action, the smartphone just displays the ECG signals on the screen, and the microcontroller transmits every 5^th^ sample of the ECG signals in order to reduce the transmission load. This 100 SPS is enough for the display on the smartphone screen. (iii) At “stop” action, there is no signal transmission.

### Android Smartphone Application

There are three threads in the application (see Supplement Figure S2): User interface (UI), Bluetooth, and real-time calculation (RTC) threads. The UI thread provides diverse options for the user, and displays the ECG signals and the estimation results. The RTC thread estimates the ischemic index, RR and TV in real-time. Finally, the Bluetooth thread communicates with the microcontroller, and receives the ECG signals.

### Animal Preparation and Data Recording

The animal studies were approved by the institutional review board and the subcommittee on research animal care at Massachusetts General Hospital. All experiments were performed in accordance with relevant guidelines and regulations.

Six male Yorkshire swine were anesthetized and instrumented in the Animal Electrophysiology Laboratory at Massachusetts General Hospital, as previously described^21^,^22^,^23^. Each animal was intubated and placed on a mechanical ventilator, and anesthesia was maintained with isoflurane. An Ohmeda anesthesia system with an Ohmeda 7800 ventilator (GE, Madison, WI) was used to control TV. A respiratory monitor (Surgivet V9004, Smiths Medical, Dublin, OH) was used as the gold standard to measure and confirm the RR throughout each respiratory intervention. This monitor has an accuracy of ± 1 breath/min. Electrodes were placed at the standard 12-lead ECG placement locations.

For the myocardial ischemia (MI) testing, percutaneous access to the femoral arteries and veins was achieved using Seldinger techniques, as previously described^21^,^24^. Regional MI was induced by balloon occlusion of the left circumflex coronary artery or the left anterior descending artery, using standard percutaneous cardiac catheterization techniques. Ischemia was validated and confirmed by hand injections of contrast into the coronary, in which case no-flow as well as electrocardiographic changes were indications of full occlusion^21^,^24^,^25^.

For the RR testing, the RR was changed from 6 to 14 breaths/min. For the apnea testing, the ventilator was suspended for up to 2 min following a few minutes of normal respiration. The ventilator remained steady for ~2 min at each new setting.

### ECG-Processing Algorithms

A software-based QRS detection algorithm was applied, to a predetermined lead, to obtain preliminary R-wave annotations. The preliminary QRS detections were refined, and abnormal beats, e.g., premature ventricular complexes and aberrantly conducted beats, were identified using a template-matching QRS alignment algorithm^21^,^22^,^23^,^24^. Briefly, for each new beat an 80 msec window centered at the peak of the QRS complex was formed from the preliminary beat detection; an isoelectric PR segment was automatically subtracted as a zero amplitude reference point (by estimating the mean voltage in a 10 msec window preceding the start of each QRS complex). A median QRS template was generated from all normal QRS complexes across the previous 31 beats and the beat was aligned to the QRS template using cross-correlation. Cross-correlation was repeated twice for each new QRS complex to ensure proper QRS alignment. A beat was considered abnormal if its correlation coefficient was less than a threshold value of 0.90, or if the preceding R-to-R interval was at least 10% shorter than the mean R-to-R interval of the previous 7 beats.

In order to estimate the ischemic index, due to variability in the ECG morphology, ECG annotations were independently determined for each body surface lead. Briefly, for each beat, initial T-wave boundaries were established using a rate-based T-wave window formula, in which the window begins 100 msec after the R-wave if the previous R-to-R interval was greater than 770 msec, 7.8% of the R-to-R interval plus 40 msec if the R-to-R interval was between 320 and 770 msec, and 65 msec if the R-to-R interval was less than 320 msec. The T-wave window ends 500 msec after the R-wave if the previous R-to-R interval was greater than 770 msec, or ends at 65% of the R-to-R interval if the previous R-to-R interval was shorter than 770 msec.

For the estimation of the ischemic index, T-wave boundaries were detected lead-by-lead by performing linear baseline adjustment across the T-wave window (using the approximate T-wave boundaries described above), squaring the T-wave, integrating the T-wave power, and determining new and more accurate T-wave boundaries at timings corresponding to 1% and 99% of the signal power respectively.

QRS boundaries were also detected employing the above method, using an initial window extending from 50 msec prior to the QRS detection point to either 80 msec after the QRS detection point or to the beginning of the T-wave, whichever was shorter.

#### Respiration Rate & Tidal Volume Estimation

The apex of the heart is stretched towards the abdomen during inspiration, and compressed towards the breast during expiration. In addition, filling and emptying of lungs changes distribution of the thoracic impedance. Therefore, respiration generates movement of the heart and change of the thoracic impedance, which cause periodic amplitude modulation of the ECG signals. We used the root mean square (RMS) values of the ECG signals to extract this periodic modulation. That is, we calculated the RMS value of each beat in an 80 msec window centered at the peak of the QRS complex; the derived RMS envelope exhibited periodic oscillation^22^,^23^.

However, in this study there are a few notable improvements in the RR and TV estimation methods: (i) a rate corresponding to a frequency of the FFT spectrum that is < 0.03 cycles/beat was considered to be an apneic event; (ii) in a prior study^23^, the RMS value of an abnormal beat was obtained using cubic spline interpolation of normal beat RMS values. In this study, we modified the RR estimation algorithm so that provided that the RR remains relatively constant (quasi-static), if there are more than 10% abnormal beats in the 32-beat window, then the corresponding RR is interpolated using the cubic spline method (please, see Supplement Figure S8).

#### Ischemia Detection

ST-segment deviation (elevation and depression) has been well established as a strong marker of myocardial ischemia^26^. The ischemic index, which has been introduced in a previous study^21^, is defined as the absolute value of the ratio of ST height to the QR amplitude. The ST height is defined as the mean amplitude calculated over the whole ST-segment above or below the isoelectric baseline, when the polarity at both ends of the ST-segment is the same. If the polarity is opposite, then the longer segment is selected for the ST height calculation.

### Statistics

Our results are presented as mean ± standard deviation of normally distributed variables, unless otherwise noted.

The Kruskal-Wallis test was used to test changes in the ischemic index from baseline to subsequent measurements, at each time bin (1 min). A statistically significant change is manifested by a p value less than 0.05. Statistical analysis was performed using MATLAB (MathWorks Inc, Natick, MA).

## Results

### Comparison of ECG Signals between the Smartphone and Prucka Cardiolab

In prior studies, we have used the Prucka Cardiolab electrophysiology system (General Electric), to record body surface ECG signals^22^,^23^. In this study, we developed a simple connector that allows the simultaneous recording of ECG signals from both Prucka Cardiolab and the Smart-Phone, from each electrode. We compared the 12 lead ECG signals acquired simultaneously from Prucka Cardiolab and the Smart-Phone. We have observed that the two signals show the same ECG morphologies in all leads (please see Supplement Figure S3).

To evaluate the noise level of cvrPhone, the noise level of every beat across all leads was calculated as the standard deviation of ECG values for 20 ms in a flat period between the T-wave end and the beginning of the following P-wave or in the middle of PR interval (see Supplement Figure S4). The noise level of cvrPhone is statistically significantly lower than the Prucka Cardiolab (Wilcoxon rank sum test, p <  = 0.001), most likely due to the DC power supply of cvrPhone.

Thereafter, we used previously developed algorithms for the estimation of the RR^23^, TV^22^ and ischemic index^21^, which were modified for the cvrPhone described above, and for which we first confirmed that their JAVA implementation for the Android was equivalent to the previously developed one in MATLAB (see Supplement, Figures S5–7).

### Respiration Rate and Tidal Volume Estimation

**Figure 2:**
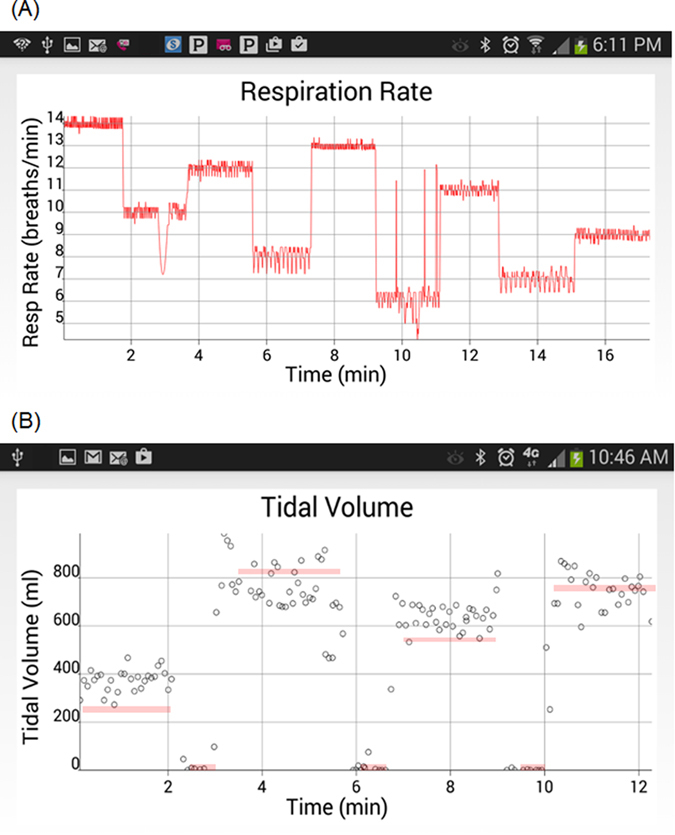
Estimation of the respiration rate (RR) and tidal volume (TV). Different RRs and TVs were obtained by adjusting the mechanical ventilator and using the reading in its display as the gold-standard. (**A**) The respiration rate was randomly changed from 14 to 10 to 12 to 8 to 13 to 6 to 11 to 7 to 9 breaths/min, while the TV was 500 ml and the heart rate was ~122 bpm. (**B**) The TV was changed from 250 to 0 to 820 to 0 to 540 to 0 to 760 ml (marked by the thick red lines), while the RR was 14 breaths/min.

We first sought to examine the ability of cvrPhone to track RR and TV changes using the reading in the mechanical ventilator’s display as the gold-standard. In Fig. 2A we present an example in which the RR was step changed randomly in the range of 6–14 breaths/min. The estimated RR values mostly show excellent agreement with the real ones (R^2^ = 0.97912). In Fig. 2B, the tidal volume was changed randomly from 0–820 ml. The estimated TV values mostly show very good agreement with the real ones (R^2^ = 0.8517).

### Apnea Evaluation

**Figure 3:**
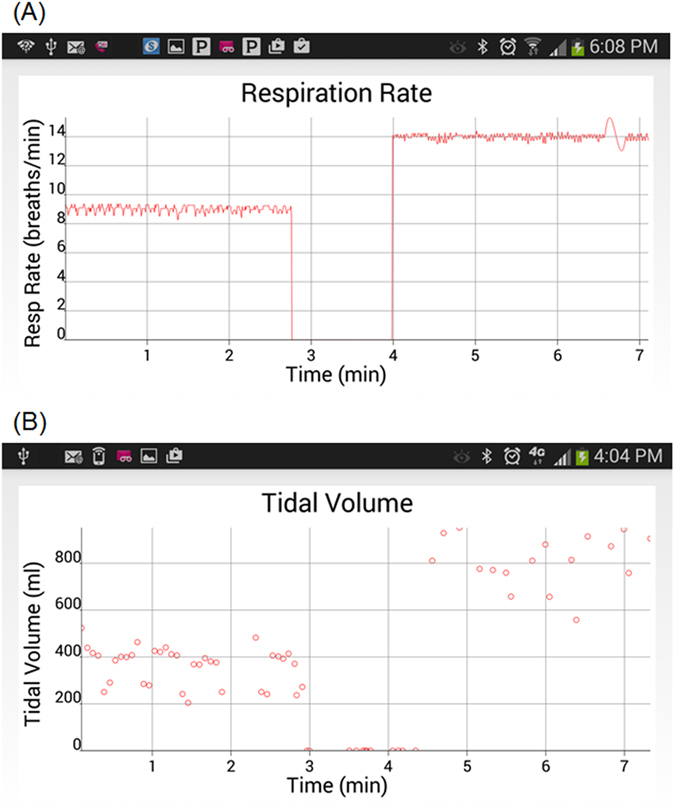
Assessment of apnea. (**A**) Estimation of the respiration rate (RR) during an apneic event. The RR was changed from 9 breaths/min to 0 breaths/min (ventilator-off) to 14 breaths/min, and the tidal volume (TV) was changed from 760 ml to 0 ml (ventilator-off) to 300 ml. (**B**) Estimation of the TV during an apneic event. The TV was 250 and 690 ml, and the RR was 14 and 6 breaths/min, before and after the apneic event, respectively. The apnea period occurs from 2 min and 53 sec to 4 min and 43 sec.

To assess the ability of cvrPhone to determine an apneic event, we suspended the ventilator for a period of ~90 sec between two normal breathing periods. Figure 3A shows the estimated RR values during an apnea test; the true RR values before and after apnea are 9 and 14 breaths/min respectively, while the estimated ones exhibit a less than one breath per min error.

We then ventured to assess the time our RR estimation algorithm needed to detect apnea (zero breaths per min), from normal breathing. We observed that, for an initial RR of 10.2 ± 3.6 bpm (n = 5), the time our algorithm needed to detect apnea was 7.9 ± 1.1 sec.

Figure 3B shows the estimated TV values during another apnea test. While RR was estimated at each heart beat, TV estimation was performed at each breathing cycle. During apnea, the estimated TV values reflect non-ventilation fluctuations of the RMS envelope, which are very small (<100 ml) compared to TV of normal breathing. The real TV values before and after the apnea are 250 and 690 ml, respectively. In this particular example, the TV estimation errors before, during, and after apnea were 114.1 ± 80.7 ml, 0 ± 0 ml, and 150.5 ± 168.1 ml, respectively. Overall, the error in tidal volume estimation (n = 5) before, during, and after apnea was −47.4 ± 259.7 ml, 20.9 ± 52.2 ml, and −86.1 ± 177.4 ml, respectively.

We again sought to assess the time our TV estimation algorithm needed to detect apnea from normal breathing. Given that we have previously reported^22^ an error in estimating the TV of ~73 ml during apnea, we used this number as a threshold to detect transition to apnea. We observed that for an initial TV 517.7 ± 222.0 ml (n = 5), the time our algorithm needed to detect apnea was 5.5 ± 2.2 sec.

### Ischemia Evaluation

**Figure 4:**
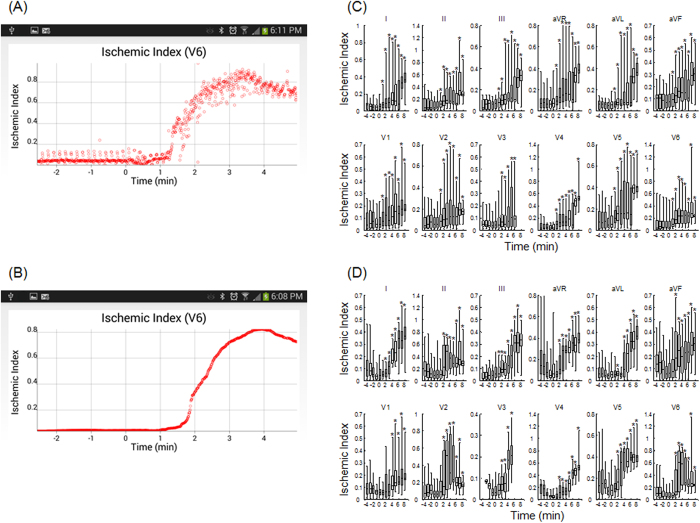
Temporal changes of the ischemic index after coronary artery occlusion. (**A**) Beat-by-beat ischemic index estimation before and after coronary artery occlusion (t > 0 min), of lead V6. (**B**) One-minute, running median ischemic index estimation. (**C**) Summary results of beat-by-beat, ischemic index estimation (n = 9 records from 6 animals). There is a statistically significant (p < 0.018) increase of the ischemic index after coronary artery occlusion. Each bar graph represents 5, 25, 50, 75 and 95 percentiles of ischemic indices beat-by-beat estimated for all animals for 1 minute time span. (**D**) Summary results of beat-by-beat, ischemic index estimation preceding ventricular tachycardia (n = 3). The ischemic index distributions that are significantly increased after occlusion compared to baseline, are indicated by an asterisk (p < 0.028).

Figure 4A shows beat-to-beat estimation results of the ischemic index from lead V6 following an ischemic event (time 0 min). One observes that the ischemic index starts increasing abruptly about 75 sec after the occlusion. In Fig. 4B we present a 1 min running median of the ischemic index values, which as expected displays a smooth curve that exhibits an ~30 sec delay with respect to the timing of the beat-by-beat ischemic index values.

Figures 4C,D show summary results of temporal changes of the ischemic index following coronary artery occlusion (time >0 min). Figure 4C includes results from 6 animals, three of which resulted in spontaneous ventricular tachycardia episodes during acute ischemia (these results are presented separately in Fig. 4D). Within two minutes from occlusion we observe (Fig. 4C) that the ischemic index, in the majority of body-surface leads, becomes significantly (p < 0.05) higher compared to baseline. With respect to the ability of each the 12 ECG leads in detecting underlying ischemia, we sought to estimate the time needed the ischemic index following coronary artery occlusion to exceed the median +3 stdev, of its baseline value. As expected, each lead exhibited a differential ability to detect the onset of ischemia, with V5 showing close to the shortest time (0.98 ± 0.87 sec) due to its proximity to the circumflex coronary artery.

In 3 MI cases (an example is shown in Fig. 4D), tachycardia developed 294 ± 166 sec after the coronary artery occlusion, which was preceded by significant increases of the ischemic index for about 1, 4 and 1 min, respectively.

**Figure 5:**
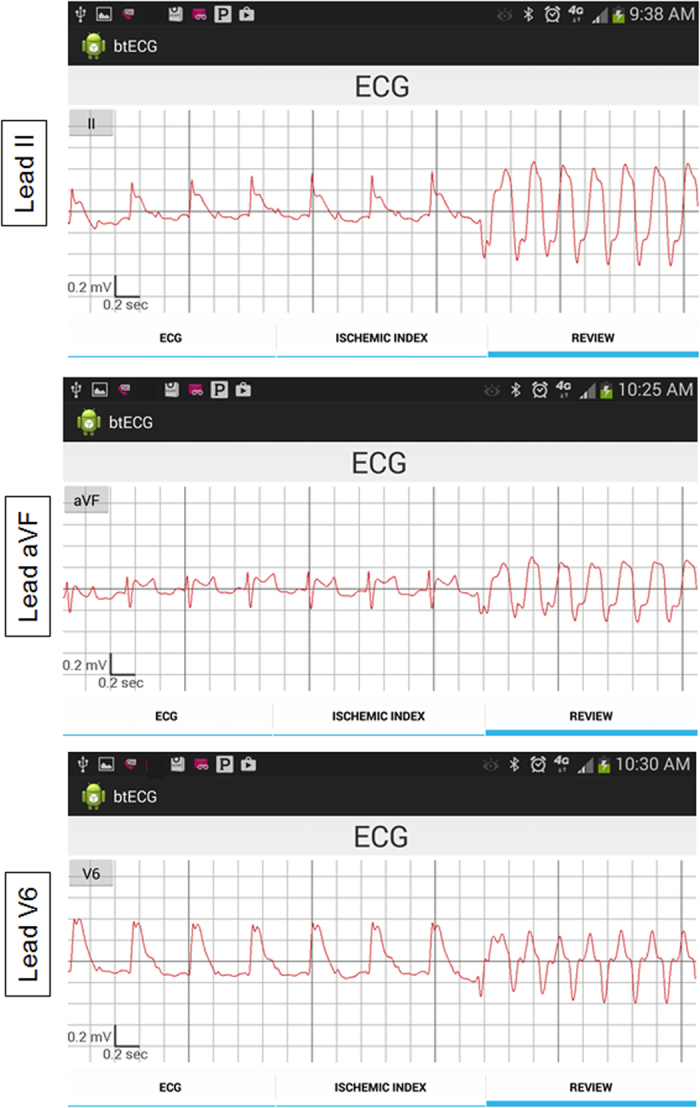
ECG signals displaying spontaneous transition to ventricular tachycardia after coronary artery occlusion, which occurred 298 sec after the occlusion.

In Fig. 5 we present ECG signals (leads II, AVF, V6) during the transition from sinus rhythm to ventricular tachycardia after coronary artery occlusion. Such information could alert both a physician and a patient and result in timely delivery of therapy and perhaps prevention of sudden cardiac death.

## Discussion

**Figure 6:**
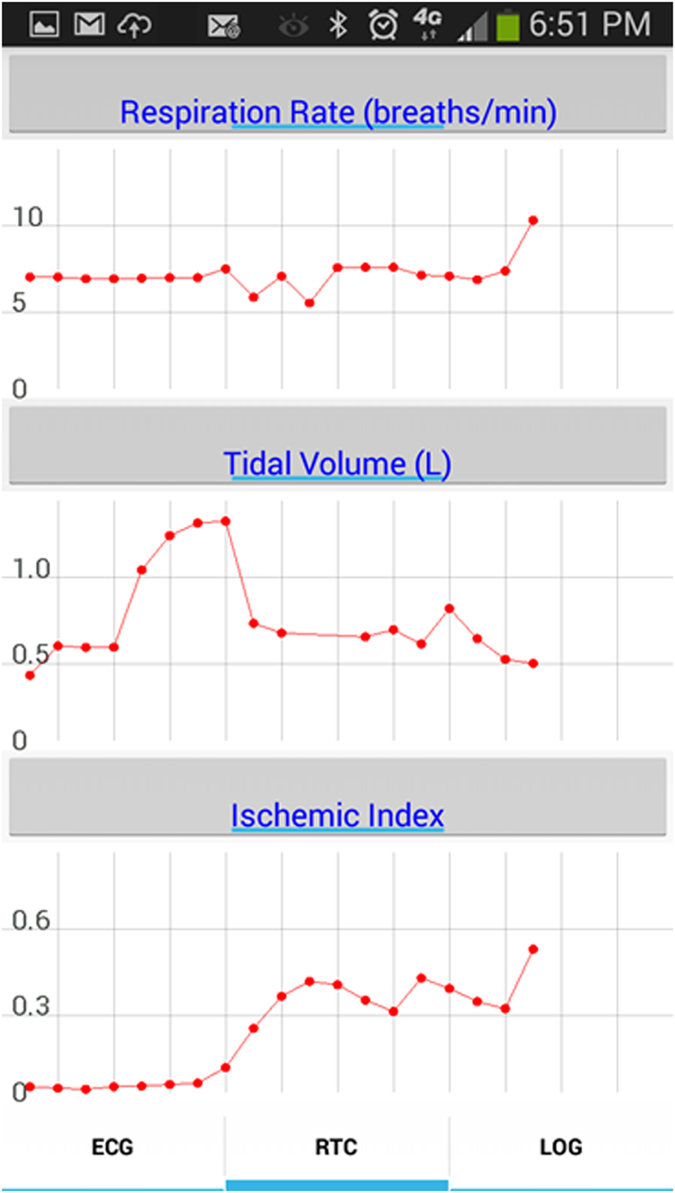
Real-time display of the respiration rate, tidal volume and ischemic index. Each red dot represents a 1 min running median estimated value obtained every 30 sec.

We have developed a smartphone-based cardio-respiratory monitoring system (Fig. 6), called the cvrPhone, in which we adopted and modified algorithms to estimate the respiration rate and tidal volume and to detect underlying ischemia that we have developed and validated in prior studies^21^,^22^,^23^.

Chronic medical conditions are becoming increasingly prevalent in the U.S., leading to increasing morbidity and mortality, as well as to increased healthcare expenditures. Developing novel methods of more efficiently diagnosing and monitoring such chronic conditions outside the hospital or clinic setting could very likely have a significant and favorable impact on both health and cost. Among patients with coronary heart disease, the ability to detect asymptomatic ischemic ECG changes could alert the patient and providers about the possibility of disease progression and impact decision making about therapeutic strategies that could potentially reduce both morbidity and mortality from ischemic heart disease. Moreover, when patients with coronary heart disease do have symptoms of chest pain, they often don’t know whether the symptoms are angina or not, and therefore are uncertain about the need to seek prompt medical attention. Indeed, for patients suffering an acute myocardial infarction, a major contributor to delays in reperfusion therapy is the interval between symptom-onset and the decision to seek care, which reflects patients lack of confidence in their own ability to recognize the symptoms of acute myocardial infarction^27^. Therefore, reliable ambulatory monitoring systems that can accurately detect the presence of myocardial ischemia and prompt patients in real time to seek appropriate medical attention could potentially improve clinical outcomes in acute coronary syndromes.

The ability of the cvrPhone to detect changes in respiratory physiology has the potential to improve the diagnosis and monitoring of CSR in patients with HF, which could, in turn, lead to better risk stratification or more timely intervention. Moreover, accurate respiratory monitoring could be used to diagnose and monitor other forms of sleep disordered breathing (SDB), such as obstructive sleep apnea, which is associated with a significant increase in cardiac morbidity and mortality^28^,^29^; importantly, that risk can be reduced through the appropriate use of continuous positive airway pressure^28^,^29^. In this study, we have shown that cvrPhone can successfully detect apneic events using the implemented RR and TV algorithms independently, with the estimated RR values being 0 breaths/min and the estimated TV values being less than 100 ml, during apnea (Fig. 3). However, even though many studies support the concept that sleep apnea in HF patients is associated with higher incidence of life-threatening nocturnal arrhythmias, the relationship between HF, sleep apnea and arrhythmias remains poorly understood. Investigation has been hampered by limited clinical data that includes both cardiac electrical activity and the respiratory status of the patient. In this regard, the extraction of the respiratory parameters from ECG signals, may be a potentially useful tool in understanding the pathophysiology of sleep apnea-induced arrhythmias.

Since RR/TV estimation and detection of underlying ischemia rely on processing ECG signals the amplitude of which depends on the orientation of the heart and the size of the torso, it is rather difficult a priori to know which leads are more likely to contribute to the accurate estimation of RR/TV or the highest sensitivity in detecting ischemia, in a random human.

Motion can produce significant artifact that may complicate the analysis of the signals. However, under conditions such that significant artifact is generated every method to estimate the respiration rate/tidal volume or assess ischemia would be significantly impacted, as well. However, we should note, that, (i) because the system is battery powered, as we have shown in the Online Supplement, the random noise level is smaller than a power-supplied commercial system; (ii) because cvrPhone communicates with the Android through Bluetooth, there are no wires that connect the ECG electrodes with the Android (as is currently performed in cardiac stress-testing), therefore motion artifact is expected to be smaller in an ambulatory patient.

### Relevance to existing technologies

In a hospital setting, measurement of RR/TV can be accomplished either directly or indirectly^13^,^14^,^15^,^16^,^17^,^18^,^19^,^20^,^30^,^31^,^32^,^33^,^34^, using specialized hardware with features that are neither practical nor convenient for the free-moving, ambulatory subjects, especially those with chronic conditions that live in under-served areas. Similarly, ECG changes manifesting underlying ischemia are measured through dedicated equipment that the patient is constantly connected with.

24- or 48-hour Holter monitoring has been the standard approach used in clinical practice to detect, characterize and document cardiac arrhythmias, but not ischemia. However, there are some inherent limitations associated with this technology: the observation period is too short to allow capturing of an infrequent arrhythmia. As a result, Holter monitors detect the arrhythmias that are responsible for symptoms, only ~10% of the time. Although the period of observation could be extended, serial Holter monitor recordings are impractical and expensive. These observations highlight the need for tools to monitor the respiratory and cardiac state of ambulatory patients with chronic conditions.

A new FDA-approved ECG patch monitor (Zio Patch), which can be worn for up to 14 days has shown improved clinical event detection compared with the conventional 24-hour Holter monitor^35^. The fact that it offers only one lead may explain its significantly lower sensitivity compared to the conventional Holter monitor. The NUVANT, a wireless arrhythmia event monitor^36^ consists of a wearable (patch) monitoring device and a portable data transmission device. Unlike the Zio Patch, which records and stores all ECG data for retrospective arrhythmia detection, the NUVANT performs real-time analysis and transmission^36^. A device aimed for individual home use is the Scanadu Scout (a hockey puck–shaped device that is held between two fingers)^37^, which is designed to generate a complete set of vital signs, including heart rate and respiratory rate and to transmit the data wirelessly to the patient’s Smart-Phone where it can be analyzed, and then transmitted to a provider.

However, the aforementioned devices^35^,^36^,^37^ cannot provide an assessment of the patient’s respiratory state, such as sleep disordered breathing. Furthermore, none of these devices is designed to determine heart rate and rhythm, by providing providing a medical grade 12-lead ECG. At the same time, since one cannot diagnose myocardial infarction associated ST-elevation without a 12-lead ECG, the US National Heart Attack Alert Program recommends that Emergency Medical Systems provide out-of hospital 12-lead ECGs to facilitate early identification of acute MI and that all advanced life-saving vehicles should be able to transmit 12-lead ECGs to the hospital^38^. Thus, it becomes imperative that the Smart-Phone based diagnostic device of the future should be capable of recording and transmitting 12-lead ECGs to the treating medical team.

## Conclusions

In summary, we have developed a smartphone-based ECG monitoring system to detect ischemia-induced ST-segment deviation and to measure the RR and TV in real-time. Our *in-vivo* swine studies have confirmed the cvrPhone’s ability to identify ST-segment changes following acute coronary artery occlusion and to detect apneic events from analysis of ECG signals only. Further clinical studies in humans will be needed to determine the accuracy and utility of such smartphone-based monitoring in the management of chronic cardiorespiratory conditions.

In the long-term, the innovation of cvrPhone involves the development and deployment of *the first* medical-grade 12-lead electrocardiographic (ECG) system that can stream ECG data in real-time to a personalized ambulatory monitoring device (such as the Smart-Phone), and for estimation of the RR/TV and minute ventilation (MV) from ECG signals without the need of specialized equipment and underlying ischemia. The *end-user (both the patient and patient-care-team)* benefits from this technology are: (i) it will extend continuous respiratory monitoring to the non-ventilated patient in a way never before possible; (ii) it will provide the ability to quickly see the association between all three respiratory metrics (RR, TV and MV) as well as heart-rate (HR) in trend graphs, assisting patients and clinicians in the immediate evaluation of the patient’s respiratory system and changes in respiratory state that can precede respiratory depression and death; (iii) it will provide the ability to quickly assess an underlying ischemic episode in a trend graph and assist patients and clinicians in determining whether immediate therapy is needed in order to prevent life threatening arrhythmias and death; (iv) it will permit the care-team to better monitor complex outpatients under their care.

## Additional Information

**How to cite this article:** Sohn, K. *et al*. cvrPhone A Novel Point-of-Care Smartphone Based System for Monitoring the Cardiac and Respiratory Systems. *Sci. Rep.* **7**, 44946; doi: 10.1038/srep44946 (2017).

**Publisher's note:** Springer Nature remains neutral with regard to jurisdictional claims in published maps and institutional affiliations.

## Supplementary Material

Supplementary Information
