# Supplementary material for: A Novel Point-of-Care Smartphone Based System for Monitoring the Cardiac and Respiratory Systems
Source: Sci Rep. 2017 Mar 22;7:44946. doi: 10.1038/srep44946 (PMC5361153; doi:10.1038/srep44946)
Supplement: Supplementary Information [file srep44946-s1.pdf]

cvrPhone: a Novel Point-of-Care Smartphone Based System for Monitoring the Cardiac and Respiratory Systems

Kwanghyun Sohn, PhD, Faisal M. Merchant, MD, Omid Sayadi, PhD, Dheeraj Puppala, MD, Rajiv Doddamani, MD, Ashish Sahani, PhD Jagmeet P Singh, MD PhD, E Kevin Heist, MD, PhD, Eric M Isselbacher, MD, Antonis A. Armoundas, PhD

The cvrPhone Hardwre and Software Implementation

The cvrPhone is composed of three commercially available parts: an ECG module PSL-ECG 12MD (Physiolab) equipped with an ADconverter ADS1298 (Texas Instruments), a microcontroller board Due (Arduino) equipped with a microcontroller AT91SAM3X8E (Atmel), and a Bluetooth module HC-05 (Guangzhou HC Information Technology), as shown in Figure S1. Figure S1 (A) shows pin connections between the three parts, and Figure S1 (B) shows the actual ECG acquisition device, assembly of the three parts. The microcontroller communicates with the AD converter by the serial peripheral interface (SPI), and with the Bluetooth module by the universal asynchronous receiver/transmitter (UART). Two parallel connected 9V PP3 batteries were used to power the Due board. The microcontroller initializes the AD converter and the Bluetooth converter for communication, and transmits digitized ECG data to the Smart-phone (Figure S1 (C)).

There are three threads in the Android application for the smartphone: User interface (UI) thread, Bluetooth thread and real-time calculation (RTC) thread (Figure S2). The UI thread consists of three panes. The first panel displays ECG signals of three selected leads, and the second pane displays real-time estimation values of respiration rate (RR), tidal volume (TV) and ischemic index. The third pane is used to record study notes. The Bluetooth thread receives ECG signals from the ECG acquisition device,and sends the signals to UI thread for display on the first pane and to RTC thread for real-time calculations. RTC thread also sends calculation results to UI thread for display on the second pane.

Comparison of ECG Signals between the Smartphone and Prucka Cardiolab

We have used the Prucka Cardiolab electrophysiology system (General Electric) to record body surface ECG signals in our prior studies [1-3]. In this study, we compared the 12 lead ECG signals acquired simultaneously at the same leads from Prucka Cardiolab and the smartphone. Figure S3 shows the same ECG morphologies in all leads between the two systems. In Figure S4, the noise levels of the smartphone system are statistically significantly lower than the Prucka Cardiolab (Wilcoxon rank sum test, $p < 0.0001$), probably due to the DC power supply of the smartphone system.

Comparison of the Respiratory Rate, Tidal Volume and Ischemic Index Estimated by the Android Application and MATLAB

The three estimated parameters in this study – RR, TV and ischemic index – are primarily based on the algorithms which have been developed in MATLAB in our previous studies [1-3]. We sought to evaluate whether each of these applications was accurately transferred to JAVA for the Android, by comparing the results of the two platforms for each application in the same ECG data (Figure S5-S7).

In Figure S5, we compare the results of the RR analysis using the data previously reported by Weiss et al [3]. It appears that the results are identical at all respiration rates. The RR difference is negligible with the overall mean ± std difference between the two platforms being $9.6\times10^{-5} \pm 9.6\times10$ breaths/min, respectively ($p=0.8469$ by Wilcoxon rank sum test of the difference).

Similarly, in Figure S6, the TV results estimated using the MATLAB and JAVA platforms and ECG data presented by Sayadi et al [2], appear identical at 0.1 ml resolution ($p=1.0$; Wilcoxon rank sum test).

Finally, in Figure S7 we present dynamic changes of ischemic index at baseline (t = 0 min) and following myocardial infarction using ECG data presented by Sayadi et al [1], and the MATLAB and JAVA platforms. The mean $\pm$ standard deviation of the difference of MATLAB and JAVA values are $-1.1101 \times 10^{-10} \pm 1.9188 \times 10^{-9}$ ($p=0.9980$ (Wilcoxon rank sum test).

Algorithm for RR Estimation

Following validation of the MATLAB and JAVA respiration rate estimation platforms, we sought to evaluate the effect of the percentage of premature ventricular contractions (PVCs) in estimating the respiration rate (RR).

In a prior study [3], the RMS amplitude for all abnormal beats was obtained from neighboring RMS values using cubic-spline interpolation. By replacing aberrant beats with interpolated points, rather than the RMS values of the average good beats, we minimized discontinuities in the RMS ratio sequence prior to spectral analysis. In the present study, we used ECG data during which the RR was quasi static,[3] to examine the effect of PVCs (in a 32-beat sequence used to estimate the RR) in estimating the RR, using two different approaches: (i) interpolation of the RMS signal, or (ii) interpolation of the RR. Figure S8 presents the RR estimation error as a function of PVC percentage within a 32-beat sequence: panel (A) presents the number of 32-beat sequences used to estimate the error (in log scale), panel (B) presents the RR estimation error as a function of PVC percentage within each 32-beat sequence, using interpolation of the RMS signal, and panel (C) presents the RR estimation error as a function of PVC percentage within each 32-beat sequence, using interpolation of the RR. One observes that, in the presence of PVCs, interpolation of the RR exhibits a smaller rate increase of the error.

Therefore, under quasi-static conditions during which the RR remains relatively constant, we have modified the algorithm so that if there are more than 10% abnormal beats in the 32-beat window then the corresponding RR is interpolated using the cubic spline method.

Algorithm for TV Estimation

Following validation of the MATLAB and JAVA TV estimating platforms, we sought to examine whether we could further improve the accuracy of the TV estimation [2].

Given that the TV estimation from ECG signals relies on respiration-induced modulation of the QRS complex amplitude, we first estimated the respiratory envelope by calculating the beat-to-beat root-mean-square of the QRS complex of each lead, as previously described [2], and estimated the percent modulation (PM) that normalized peak-to-peak amplitude of respiratory envelope, as a surrogate for TV, where PM is defined as 100 (%) × (max envelope – min envelope)/(max envelope + min envelope)/2 of each cycle of the respiratory envelope. In our prior study, we used the maximum PM value among all leads to estimate the TV at each cycle of respiratory envelope and developed a model that provides the TV from PM, as follows: $TV = a \times PM + b$, where coefficients a and b were derived from a least square regression analysis based on our swine studies [2].

In the present study, we applied the least square regression analysis using six different methods (including the previously used maximum PM method), aiming to improve the accuracy of TV estimation from body surface ECG signals. The six different methods can be classified with respect to the optimization parameter:

(i) the percent modulation, PM or

(ii) the peak difference (max envelope – min envelope), PD

The first method selects the lead with the median PM, and uses the PM for TV estimation. The second method selects the lead with the maximum PM, and uses the PM for TV estimation (as previously described [2]). The third and fourth methods are identical to the first and second ones respectively, but this time the PD is used instead of the PM. The fifth method selects the lead with maximum PD, but uses the PM for TV estimation. And, the sixth method selects the lead with maximum PM, but uses the PD for TV estimation.

We employed previously reported [2] ECG data (n=10) to obtain the slope and the intercept for each of the six different methods respectively, and estimated the TV (TV = $a \times$ PM(PD) + b), using the least square analysis [2]. We then compared the error between the estimated and the true TV for each of the six methods. Figure S9 shows the normalized (by the true TV) mean squared error (MSE) and the coefficient of variation (CV), defined as σ/μ; where, σ and μ, are the standard deviation and the mean respectively of the estimated TV across animals. Although for the TV estimation, ECG signals were acquired at 0, 250, 500 and 750 ml TVs, 0 ml was excluded in Figure S9, because MSE cannot be normalized by 0 ml and CV cannot be defined at 0 ml. On average, over the three TVs, the first method (median PM) exhibits the smallest NMSE and CV values (Figure S9, panels (B) and (D)), and this is the method that we used for the TV estimation in this study.

Changes of the Ischemic Index After Coronary Artery Occlusion

Figure S10 shows additional ischemic index estimation results, similar to those presented in Figure 4 of the manuscript: (i) Beat-by-beat ischemic index estimation before and after coronary artery occlusion (t>0 min), of leads II (A) and aVF (C), as well as one-minute, running median ischemic index estimation, of leads II (B) and aVF (D).

References

1. Sayadi O, Puppala D, Ishaque N, Doddamani R, Merchant FM, Barrett C, Singh JP, Heist EK, Mela T, Martinez JP, Laguna P, Armoundas AA. A novel method to capture the onset of dynamic electrocardiographic ischemic changes and its implications to arrhythmia susceptibility. *J Am Heart Assoc*. 2014;3
2. Sayadi O, Weiss EH, Merchant FM, Puppala D, Armoundas AA. An optimized method for estimating the tidal volume from intracardiac or body surface electrocardiographic signals: Implications for estimating minute ventilation. *Am J Physiol Heart Circ Physiol*. 2014;307:H426-436
3. Weiss EH, Sayadi O, Ramaswamy P, Merchant FM, Sajja N, Foley L, Laferriere S, Armoundas AA. An optimized method for the estimation of the respiratory rate from electrocardiographic signals: Implications for estimating minute ventilation. *Am J Physiol Heart Circ Physiol*. 2014;307:H437-447

(A)

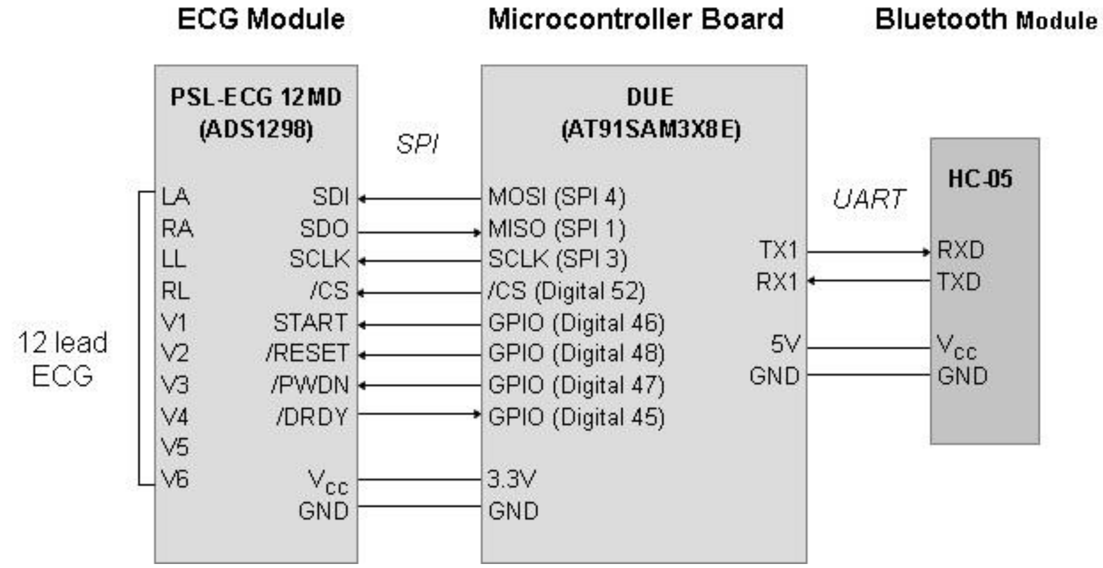
ECG Module
Microcontroller Board
Bluetooth Module
PSL-ECG 12MD
(ADS1298)
SPI
DUE
(AT91SAM3X8E)
UART
HC-05
LA
RA
LL
RL
V1
V2
V3
V4
V5
V6
12 lead
ECG
SDI
SDO
SCLK
/CS
START
/RESET
/PWDN
/DRDY
Vcc
GND
MOSI (SPI 4)
MISO (SPI 1)
SCLK (SPI 3)
/CS (Digital 52)
GPIO (Digital 46)
GPIO (Digital 48)
GPIO (Digital 47)
GPIO (Digital 45)
3.3V
GND
TX1
RX1
5V
GND
RXD
TXD
Vcc
GND

(B)

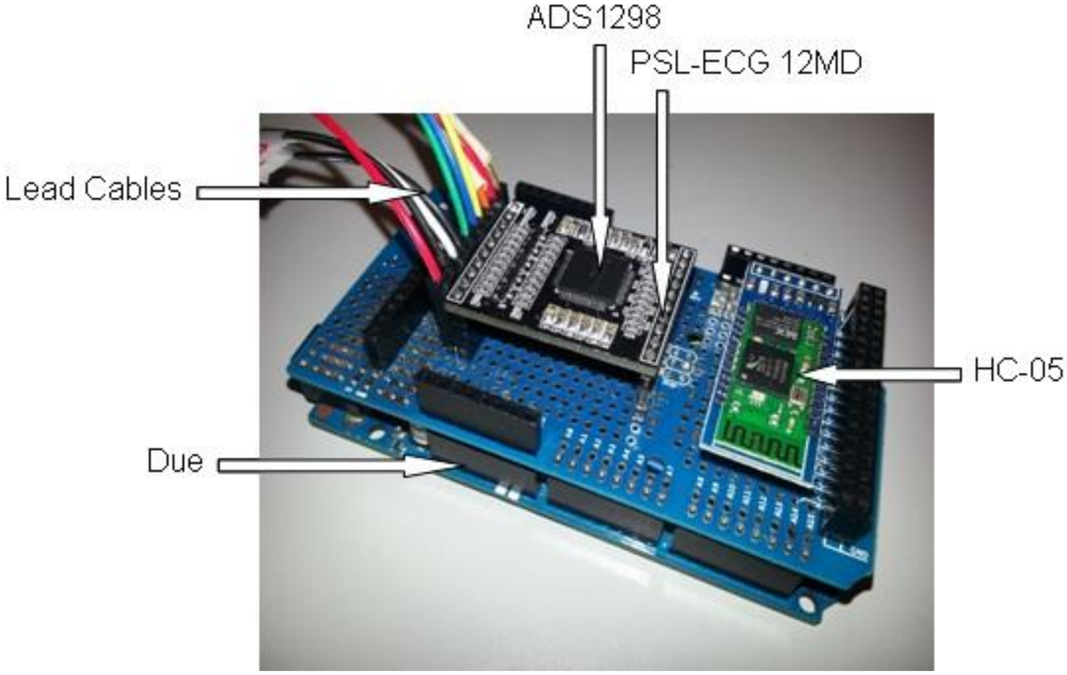
ADS1298
PSL-ECG 12MD
Lead Cables
HC-05
Due

(C)

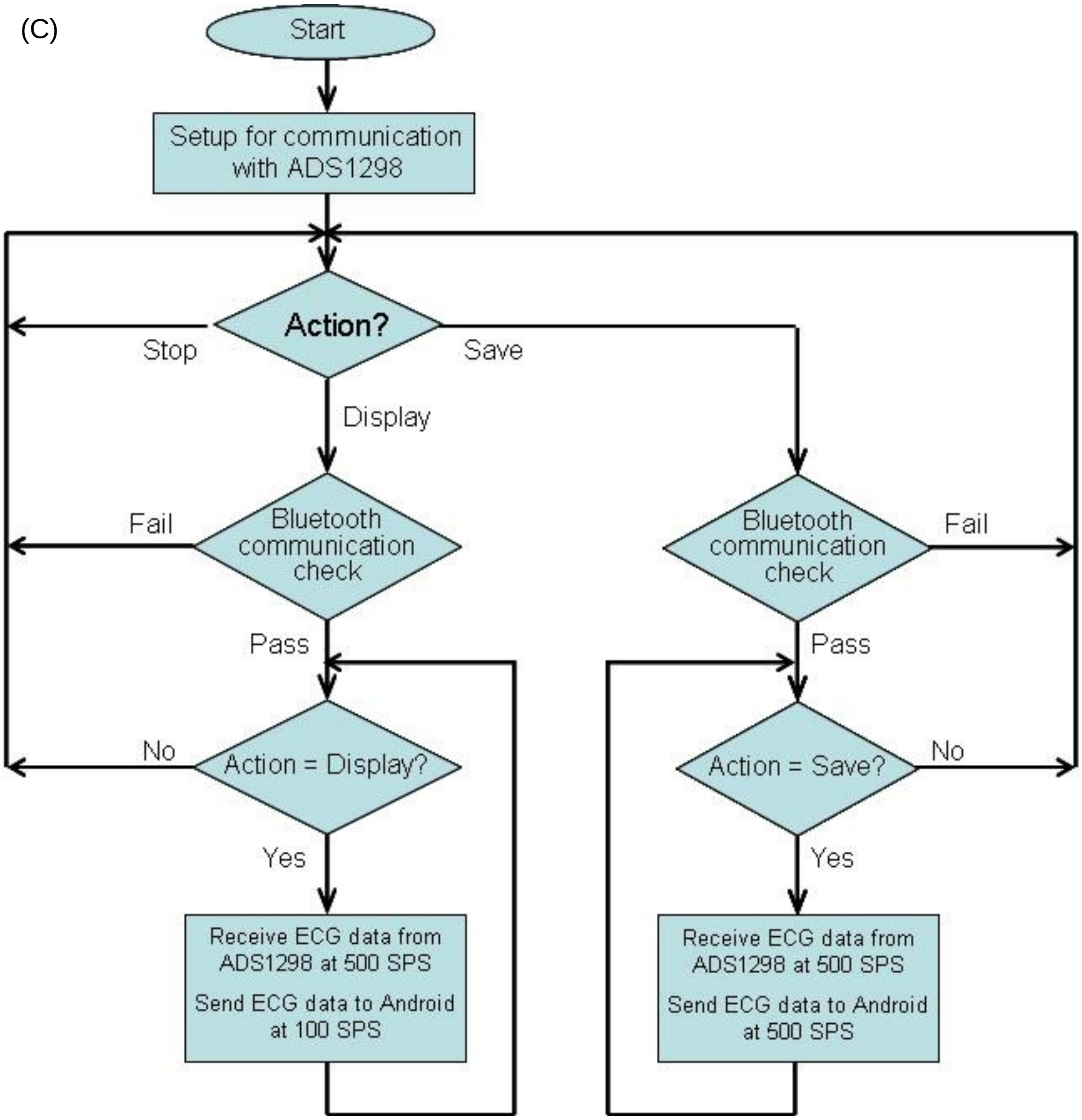

Figure S1. The cvrPhone. The cvrPhone is composed of three commercially available parts: an ECG module PSL-ECG 12MD (Physiolab), a microcontroller board Due (Arduino) and a Bluetooth module HC-05 (Guangzhou HC Information Technology). The ECG module includes an AD converter ADS1298 (Texas Instruments), and the microcontroller board which includes a microcontroller AT91SAM3X8E (Atmel). (A) Pin connections between the three parts. (B) Picture of the hardware. (C) Block-diagram of the microcontroller embedded software. The microcontroller sends ECG data to the smartphone according to user’s request.

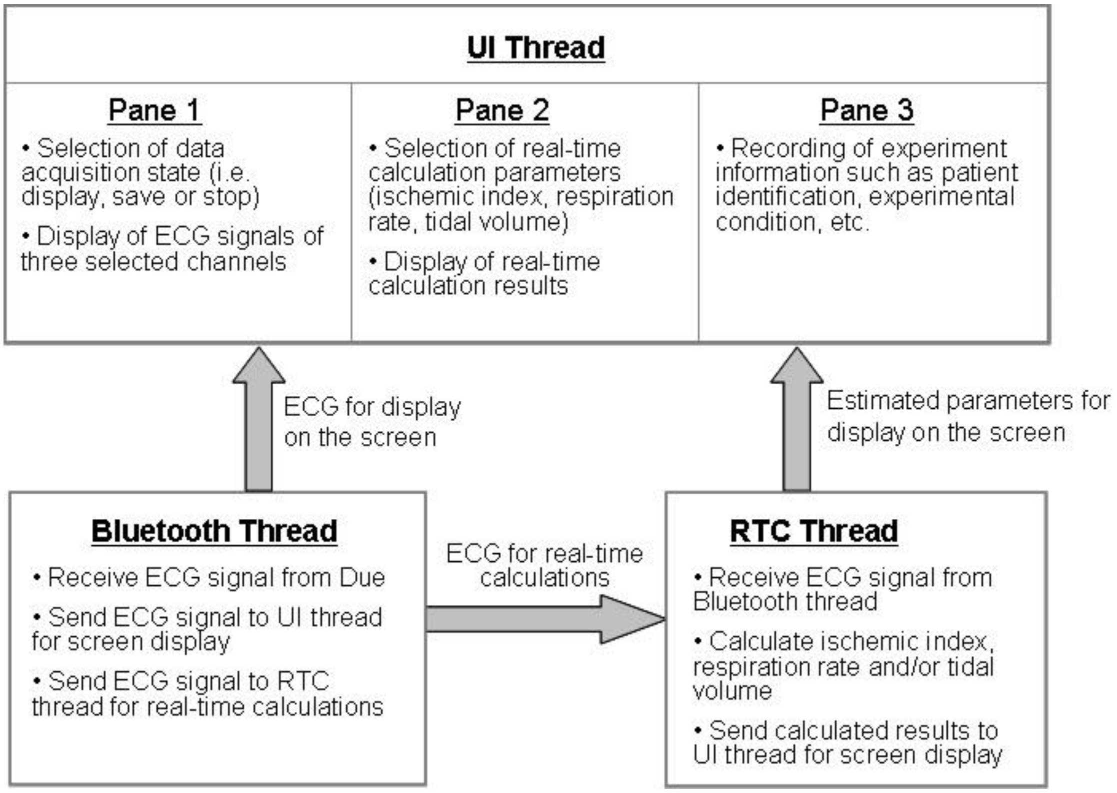

Figure S2. Block-diagram of the threads in the Android application. There are three threads in the Android application: user interface (UI) thread, Bluetooth thread and real-time calculation (RTC) thread. UI thread provides users with operation options, and displays ECG signals and real-time calculation results. Bluetooth thread receives ECG signal from the ECG acquisition device and sends it to UI thread for display and to RTC thread for real-time calculations. RTC thread estimates ischemic index, respiration rate and tidal volume in real-time, and sends the estimated values to UI thread for display.

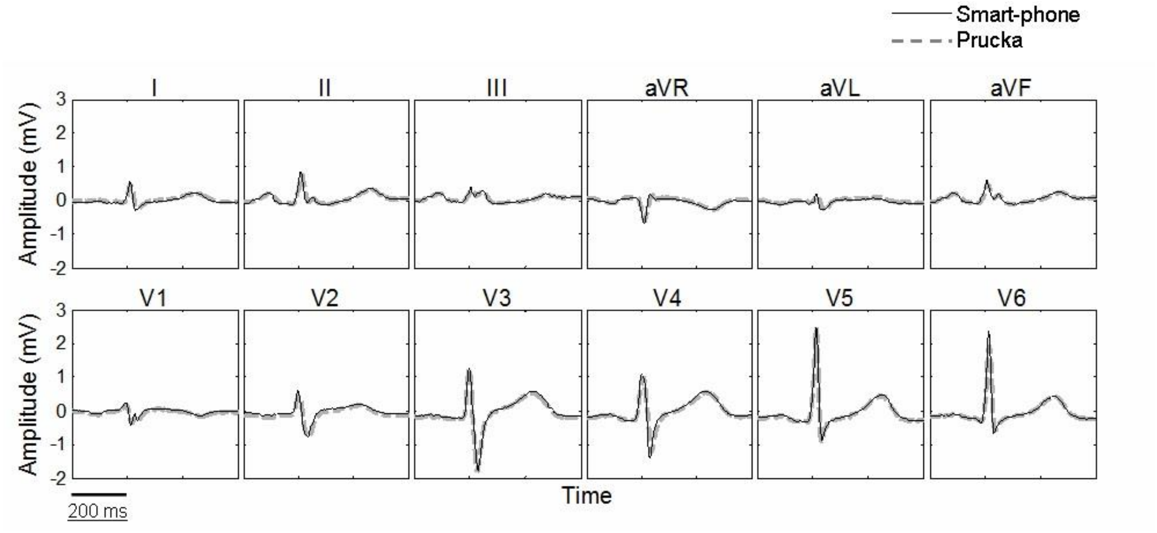

Figure S3. ECG signals acquired by the smart-phone based system (solid line) and the Prucka Cardiolab electrophysiology system (dashed line) that has been used in our animal studies before. ECG signals were acquired by both systems simultaneously from the same electrodes. The ECG module for the smart-phone system is 'PSL-ECG 12MD' (Physiolab) equipped with an AD converter 'ADS1298' (Texas Instruments).

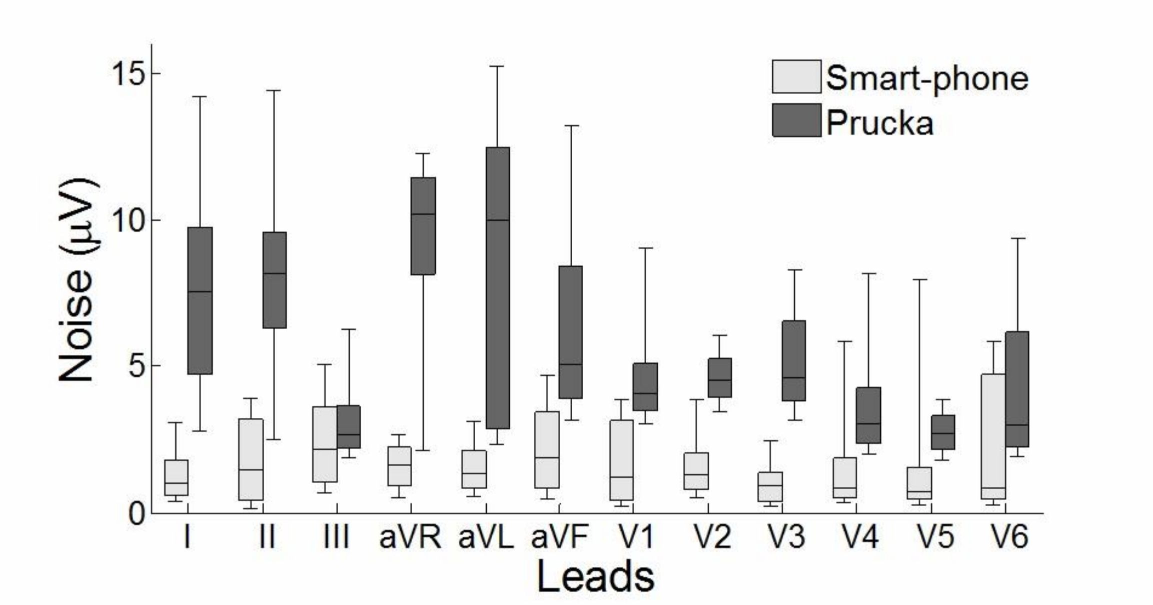

Figure S4. Noise level comparison of ECG signals acquired by the smart-phone system (light gray bars) and the Prucka system (dark gray bars). The noise level of each beat was calculated as the standard deviation in a 20 ms period between the end of the T-wave and the beginning of following P-wave or in the middle of the PR interval. Each bar graph was calculated from 2,500 beats recorded from 5 swine, 500 beats per swine. The five nodes at each bar correspond to the values of 10%, 25%, 50%, 75% and 90%. For all 12 leads, the smart-phone system exhibited significantly lower noise levels compared with the Prucka system ($p<0.0001$; Wilcoxon rank sum test).

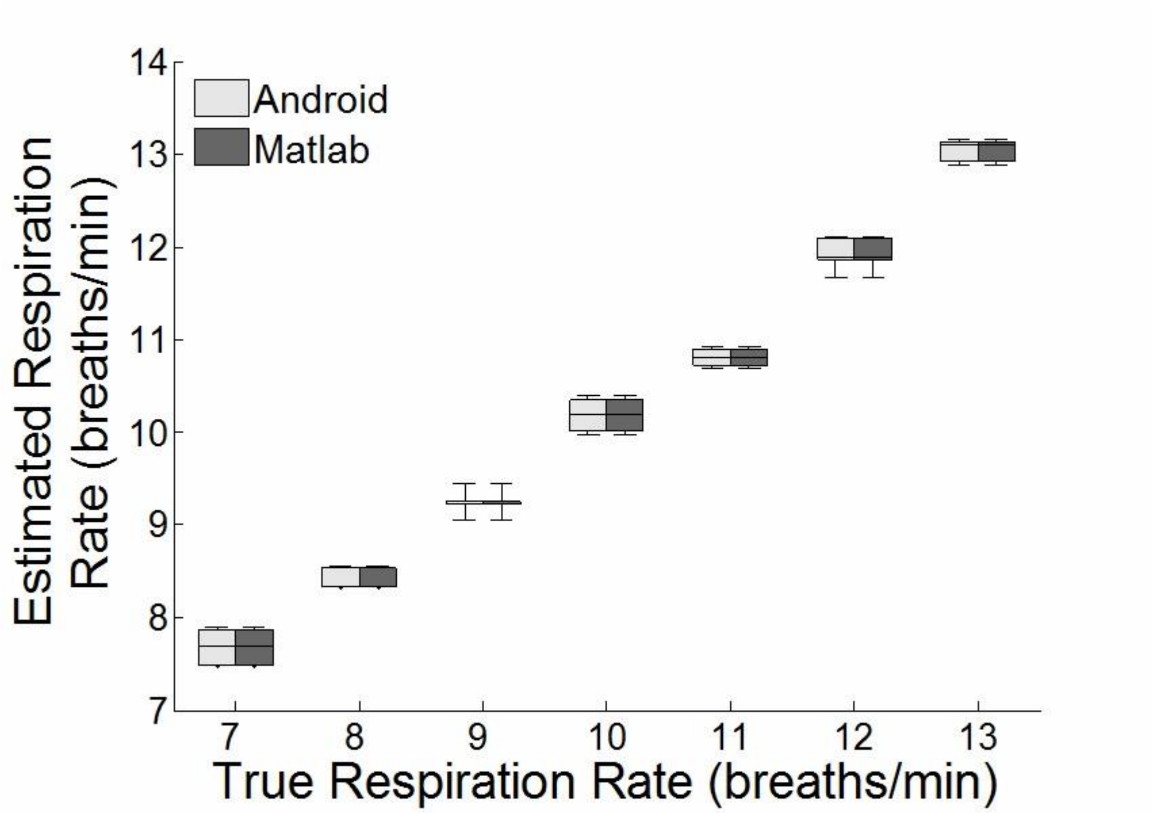

Figure S5. Comparison of the respiration rate calculated by the Android application (light gray bars) and the MATLAB code (dark gray bars). The same ECG data were used for both calculations [3]. The five nodes at each bar correspond to the values of 10%, 25%, 50%, 75% and 90%. The mean and standard deviation of the difference between Android and MATLAB values are $9.6449 \times 10^{-5} \pm 9.6175 \times 10^{-4}$ breaths/min); (p=0.8469; Wilcoxon rank sum test).

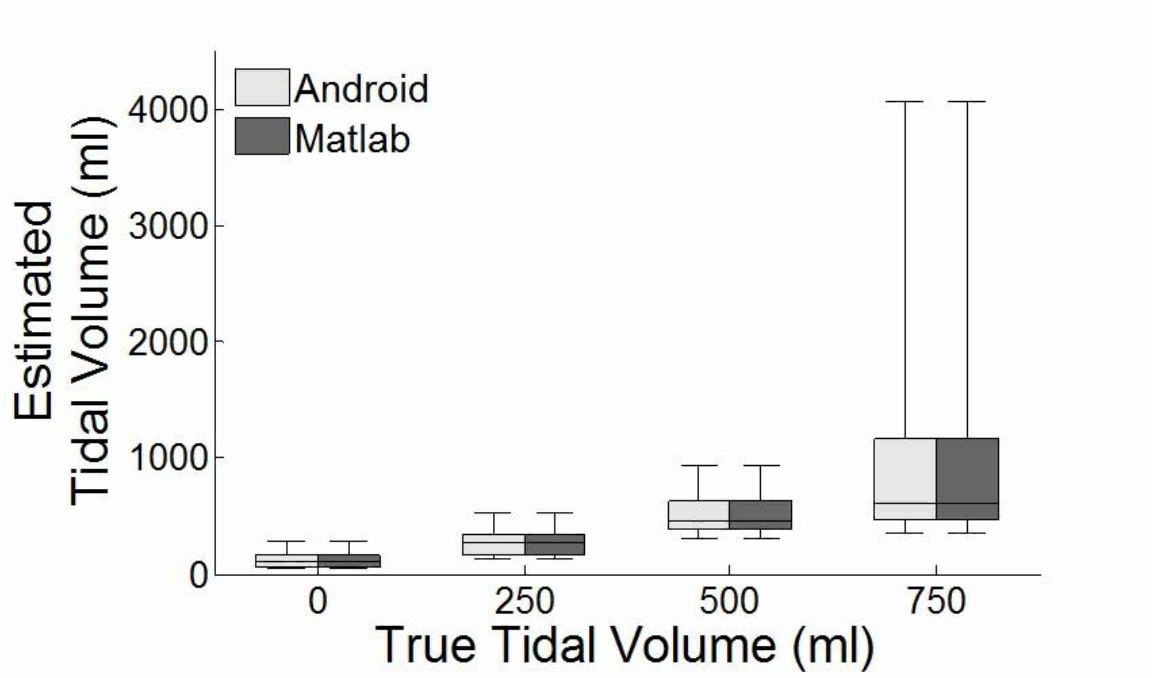

Figure S6. Comparison of the tidal volume calculated by the Android application (light gray bars) and the MATLAB code (dark gray bars). The same ECG data were used for both calculations [2]. There are 10 estimation values at each bar plot. The five nodes at each bar correspond to the values of 10%, 25%, 50%, 75% and 90%. The values from Android and MATLAB are identical at 0.1 ml resolution (p=1.0; Wilcoxon rank sum test).

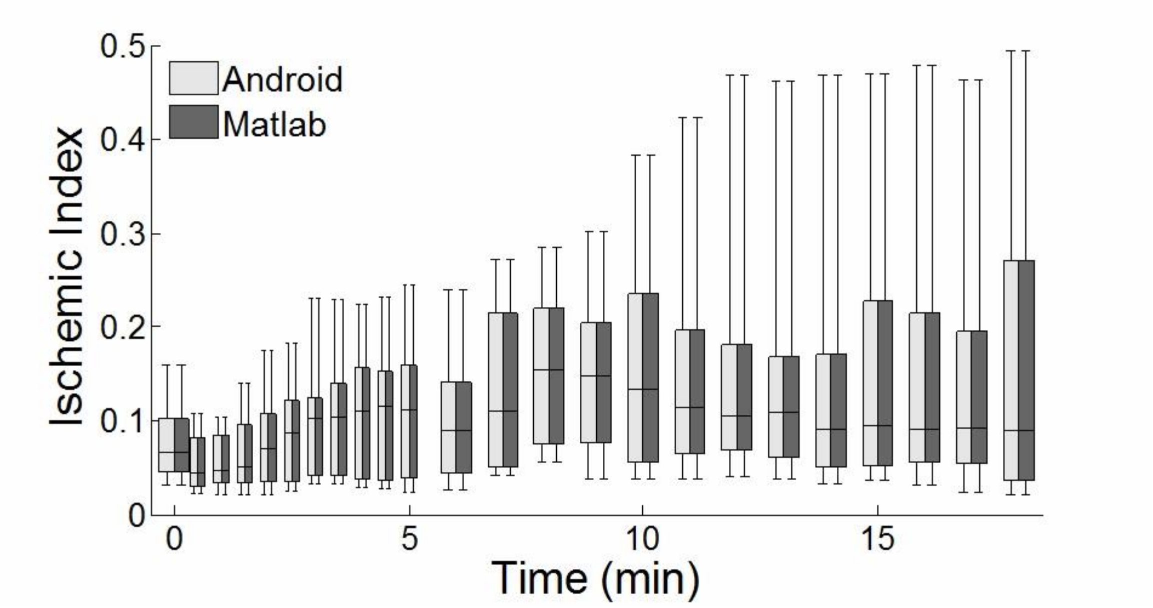

Figure S7. Comparison of two ischemic indices calculated by the Android application (left side light gray bars) and the corresponding MATLAB code (right side dark gray bars). The ECG signal was obtained from 14 swine, and the same ECG data was used for both calculations [1]. The graph shows dynamic changes of the ischemic index at baseline (t = 0 min) and following myocardial infarction. For each time bin, the distribution of ischemic index is averaged for all study subjects. The time bin width is 1 minute for baseline, 30 seconds for the first 5 minutes following balloon occlusion, and 1 minute for 6 to 18 minutes after occlusion. The five nodes at each bar correspond to the values of 10%, 25%, 50%, 75% and 90%. The mean $\pm$ standard deviation of the difference of Android and MATLAB values are $-1.1101 \times 10^{-10} \pm 1.9188 \times 10^{-9}$ (p=0.9980 (Wilcoxon rank sum test).

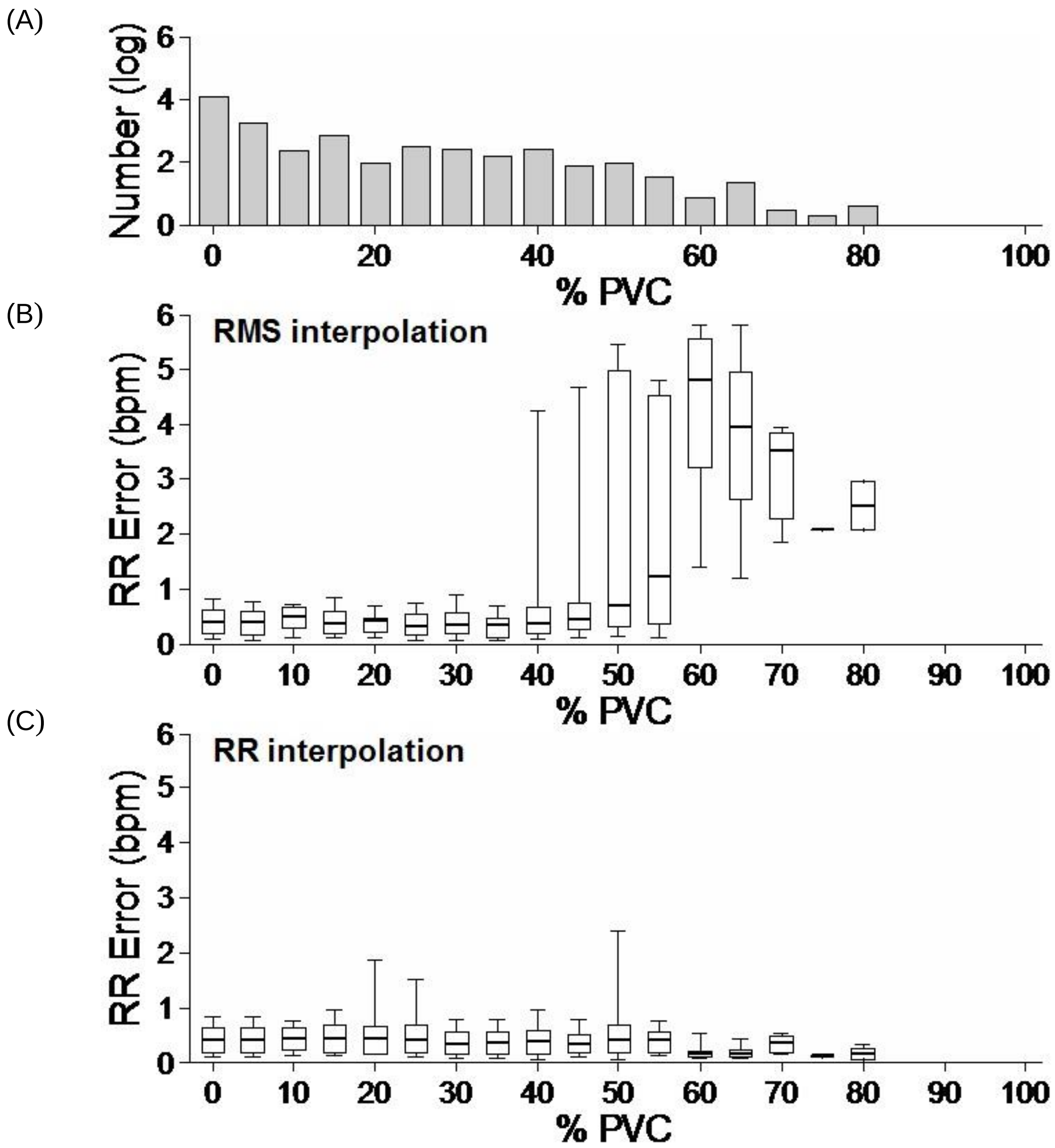

Figure S8. The respiration rate estimation error as a function of PVC percentage within a 32-beat sequence. (A) The number of 32-beat sequences used to estimate the error is shown on the top (in log scale). (B) the respiration rate estimation error as a function of PVC percentage within each 32-beat sequence, using interpolation of the RMS signal. (C) the respiration rate estimation error as a function of PVC percentage within each 32-beat sequence, using interpolation of the respiration rate. One observes that, in the presence of PVCs, interpolation of the RR exhibits a smaller error increase rate.

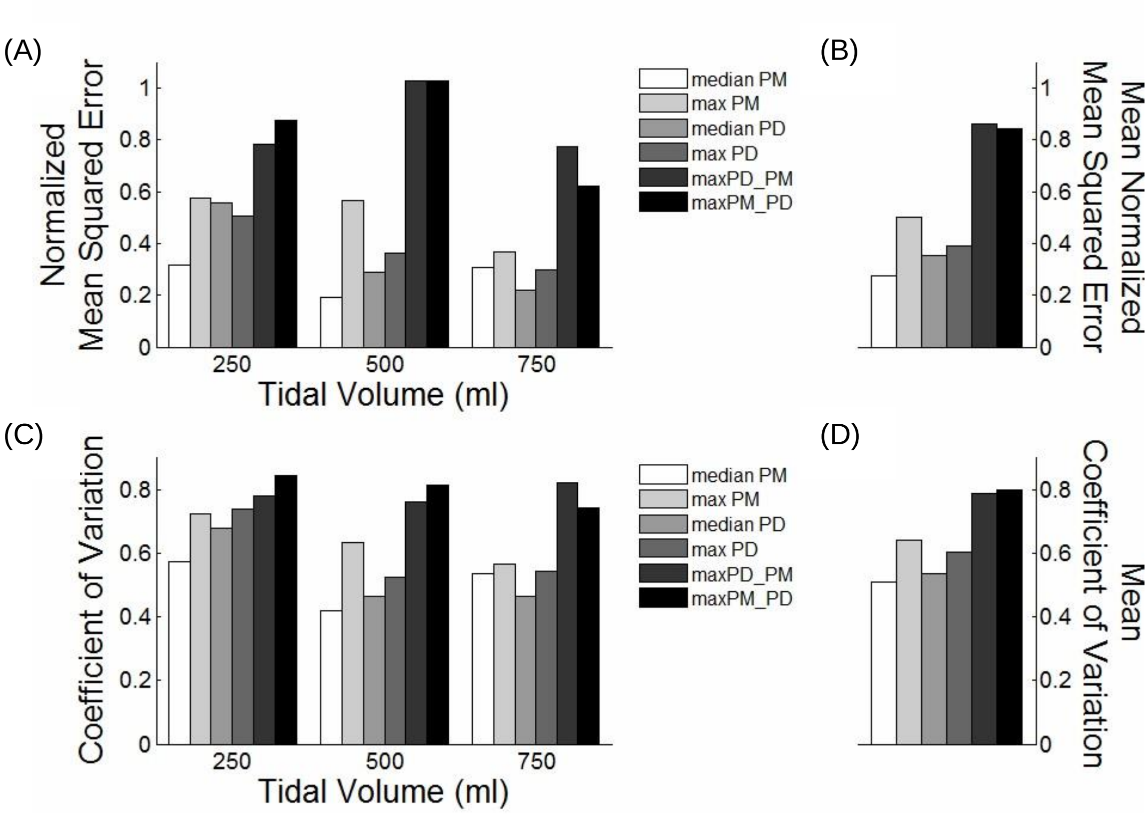

Figure S9. Normalized (by the true TV value) mean squared error (NMSE) in panels (A-B) and coefficient of variation (CV) in panels (C-D) of six different TV estimation methods. NMSE (A) and CV (C) values of the six methods are displayed at three different TV values, while average NMSE (B) and CV (D) values at the three different TVs are average at each method on the left panels. There are two parameters used for TV estimation: percent modulation (PM) and peak difference (PD). The six different TV estimation methods can be classified by the lead and the parameter that is selected for TV estimation, as: (i) the lead with median PM is selected and the PM is used for TV estimation; (ii) the lead with maximum PM is selected and the PM is used for TV estimation; (iii) the lead with median PD is selected and the PD is used for TV estimation. Max PD; (iv) the lead with maximum PD is selected and the PD is used for TV estimation; (v) the lead with maximum PD is selected and PM of the lead is used for TV estimation; (vi) the lead with maximum PM is selected and PD of the lead is used for TV estimation.

(A)

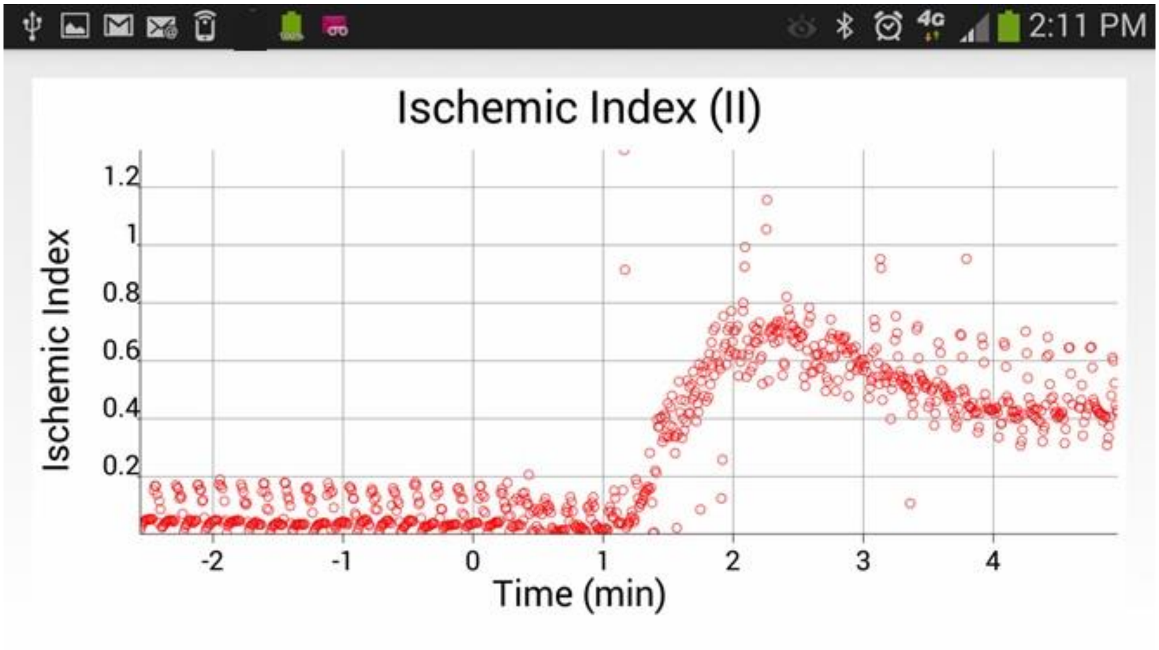
2:11 PM
Ischemic Index (II)
Ischemic Index
1.2
1
0.8
0.6
0.4
0.2
-2
-1
0
1
2
3
4
Time (min)

(B)

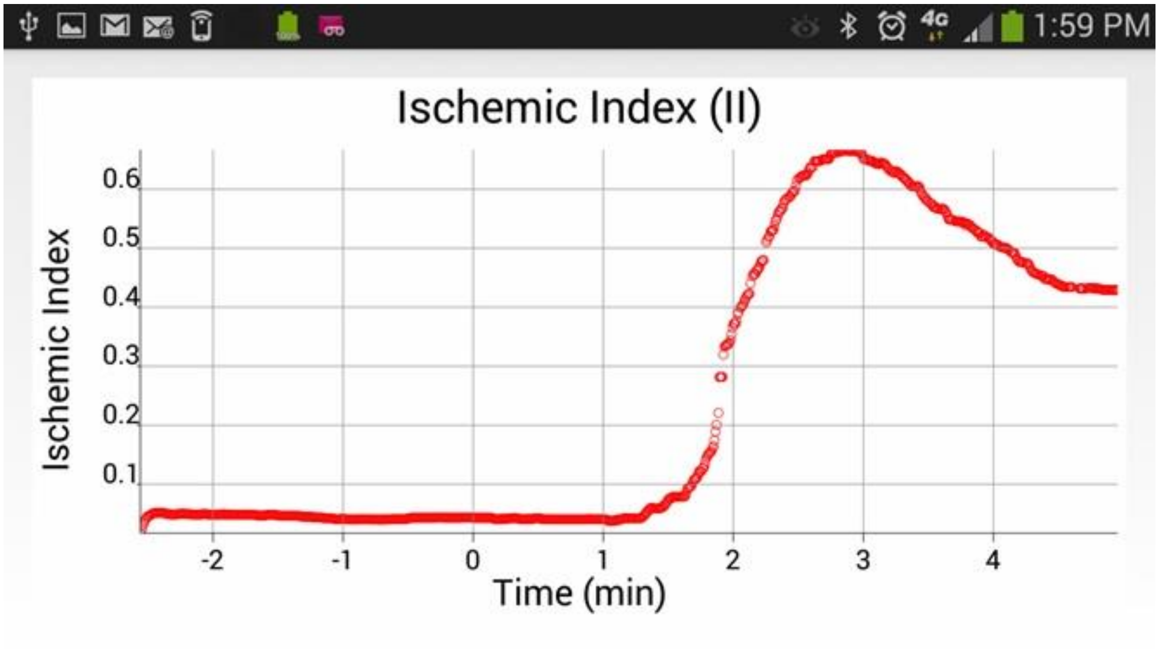
1:59 PM
Ischemic Index (II)
Ischemic Index
0.6
0.5
0.4
0.3
0.2
0.1
-2
-1
0
1
2
3
4
Time (min)

(C)

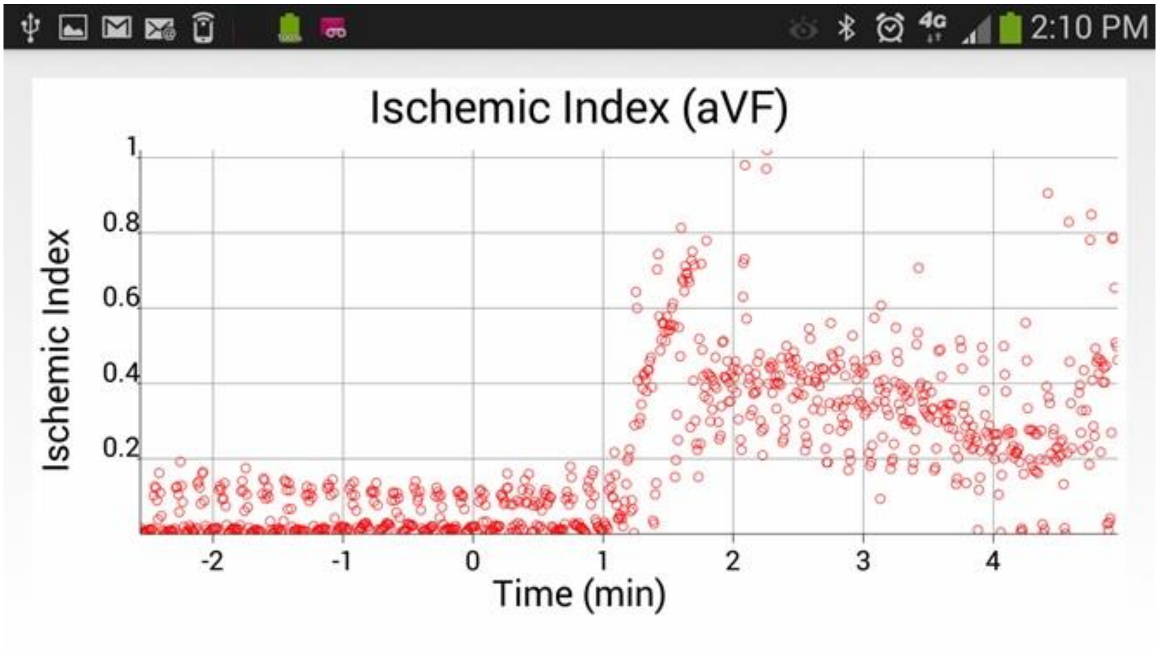

(D)

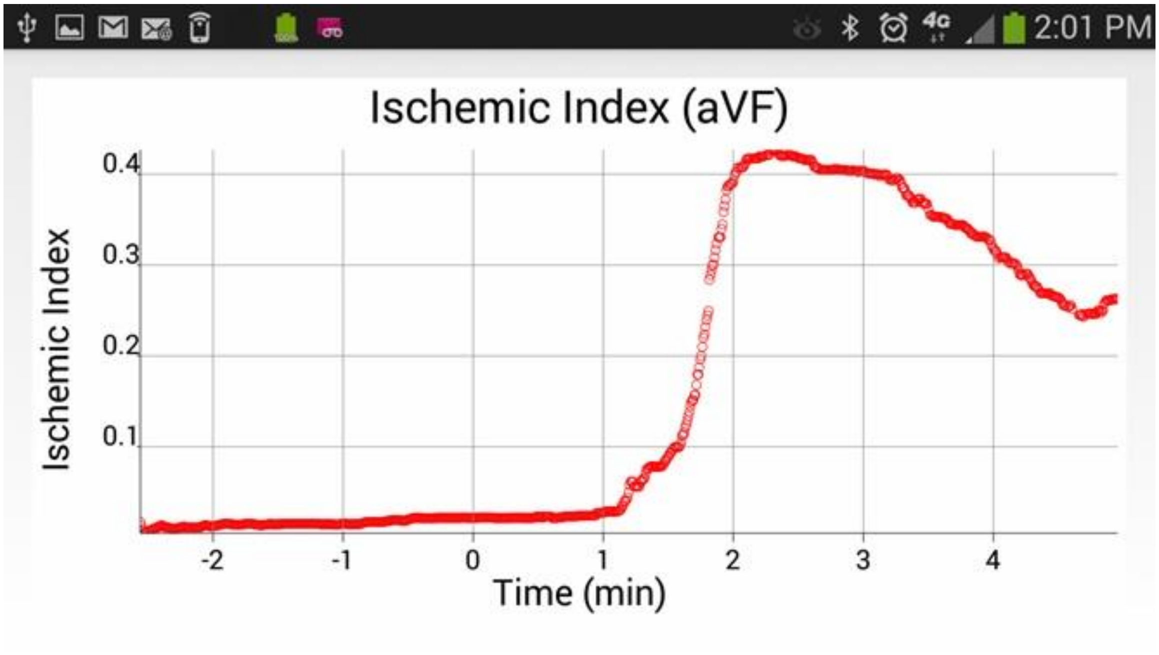

Figure S10. Changes of ischemic index after coronary artery occlusion, as described in Figure 4 of the manuscript. Beat-by-beat ischemic index estimation before and after coronary artery occlusion (t>0 min), of lead II (A) and aVF (C). One-minute, running median ischemic index estimation, of lead II (B) and aVF (D).
